# *Apis mellifera* octopamine receptor 1 (AmOA1) expression in antennal lobe networks of the honey bee (*Apis mellifera*) and fruit fly (*Drosophila melanogaster*)

**DOI:** 10.3389/fnsys.2013.00070

**Published:** 2013-10-25

**Authors:** Irina T. Sinakevitch, Adrian N. Smith, Fernando Locatelli, Ramon Huerta, Maxim Bazhenov, Brian H. Smith

**Affiliations:** ^1^School of Life Sciences, Arizona State UniversityTempe, AZ, USA; ^2^Mathematical, Computational and Modeling Sciences Center, Arizona State UniversityTempe, AZ, USA; ^3^Laboratorio de Neurobiología de la Memoria, Departamento de Fisiología, Biología Molecular y Celular, Facultad de Ciencias Exactas y Naturales, IFIByNE CONICET, Universidad de Buenos AiresBuenos Aires, Argentina; ^4^BioCircuits Institute, University of California San DiegoLa Jolla CA, USA; ^5^Department of Cell Biology and Neuroscience, University of CaliforniaRiverside, CA, USA

**Keywords:** biogenic amine receptors, G-protein receptors, octopamine, learning and plasticity, olfactory pathways

## Abstract

Octopamine (OA) underlies reinforcement during appetitive conditioning in the honey bee and fruit fly, acting via different subtypes of receptors. Recently, antibodies raised against a peptide sequence of one honey bee OA receptor, AmOA1, were used to study the distribution of these receptors in the honey bee brain (Sinakevitch et al., 2011). These antibodies also recognize an isoform of the AmOA1 ortholog in the fruit fly (OAMB, mushroom body OA receptor). Here we describe in detail the distribution of AmOA1 receptors in different types of neurons in the honey bee and fruit fly antennal lobes. We integrate this information into a detailed anatomical analysis of olfactory receptor neurons (ORNs), uni- and multi-glomerular projection neurons (uPNs, and mPNs) and local interneurons (LNs) in glomeruli of the antennal lobe. These neurons were revealed by dye injection into the antennal nerve, antennal lobe, medial and lateral antenno-protocerbral tracts (m-APT and l-APT), and lateral protocerebral lobe (LPL) by use of labeled cell lines in the fruit fly or by staining with anti-GABA. We found that ORN receptor terminals and uPNs largely do not show immunostaining for AmOA1. About seventeen GABAergic mPNs leave the antennal lobe through the ml-APT and branch into the LPL. Many, but not all, mPNs show staining for AmOA1. AmOA1 receptors are also in glomeruli on GABAergic processes associated with LNs. The data suggest that in both species one important action of OA in the antennal lobe involves modulation of different types of inhibitory neurons via AmOA1 receptors. We integrated this new information into a model of circuitry within glomeruli of the antennal lobes of these species.

## Introduction

Many studies have demonstrated that honey bees (*Apis mellifera*) and fruit flies (*Drosophila melanogaster*) can associate odors with food reinforcement (Menzel and Muller, 1996; Page et al., 1998; Scheiner et al., 2001; Keene and Waddell, 2007). These studies used sucrose reinforcement as a means of conditioning animals to respond to and discriminate among odors (Duerr and Quinn, 1982; Menzel and Muller, 1996; Menzel et al., 1999; Scheiner, 2004; Scheiner et al., 2004). Based on these learning capabilities, sensory information about sucrose reinforcement should be represented in some way by neural circuitry in honey bee and fruit fly brains (Acevespina et al., 1983; Wright et al., 2007; Engel and Wu, 2009; Cevik and Erden, 2012). Many different areas of these brains receive input from a set of ventral unpaired median (VUM) neurons with cell bodies located on the ventral midline of maxillary and mandibular neuromeres in the subesophageal ganglion (Kreissl et al., 1994; Sinakevitch et al., 2005; Sinakevitch and Strausfeld, 2006; Schröter et al., 2007; Busch et al., 2009). When stimulated, VUM neurons release the biogenic amine octopamine (OA) broadly throughout areas of the brain that are important for learning associations between many different types of stimuli, including odors and food rewards (Hammer, 1993; Hammer and Menzel, 1998).

**Figure 1:**
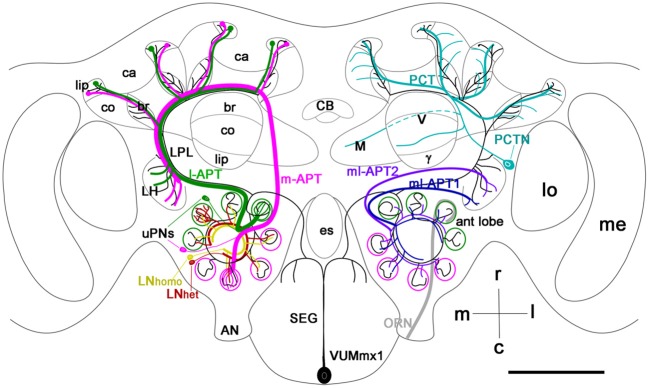
**Schematic view of the main olfactory pathways in the honey bee brain (based on Fonta et al., 1993; Abel et al., 2001; Strausfeld, 2002; Sinakevitch et al., 2005, 2011; Kelber et al., 2006; Kirschner et al., 2006; Schröter et al., 2007; Girardin et al., 2013).** Olfactory Receptor Neuron (ORN) axons from the antenna enter through the antennal nerve (AN) into the antennal lobe (ant lobe) and converge onto the outer cortex of glomeruli. Each glomerulus is innervated by processes of several types of neurons. Uniglomerular projection neurons (uPNs magenta and green) have dendritic branches in a single glomerulus and send axons to higher-order brain centers such as the MB calyces (ca), lateral protocerbral lobe (LPL), and lateral horn (LH). There are two uniglomerular antenno-protocerebral tracts, the l-APT (green) and m-APT (magenta), which reflect the segregation of glomeruli into rostral (l-APT) and caudal (m-APT) hemispherical clusters. Kenyon cells (not shown in the figure) are the intrinsic cells that make up the MB. Kenyon cell dendrites form pairs of calyces (ca) with specific zones that receive different types of inputs, the lip, collar (co), and basal ring (br). The lip and br are innervated by uPN axons. The Kenyon cell axons project ventrally and split to form medal (M), vertical (V), and γ lobes, which are the main output regions of the MB. Protocerebral tract neurons (PCTN) receive inputs in the lobes and provide GABAergic feedback to the calyces. Multiglomerular PNs project axons via the medio-lateral protocerebral tracts (ml-APT 1,2) to the LPL and LH. At least two types of local interneurons (LN) interconnect glomeruli within the AL, homogeneous LN_homo_ (yellow), and heterogeneous LN_het_ (red). Ventral unpaired median neurons (VUM) have cell bodies in the maxillary (VUMmx1 is shown) and mandibullar neuromeres of the subesophageal (SEG) ganglion and connect gustatory processing in the SEG to all antennal lobe glomeruli, the LPL, LH, and MB calyces. CB, central body; M, medial lobe; V, vertical lobe; γ, gamma lobe; lo, lobula; me, medulla; m, median; l, lateral; r, rostral; c, caudal. Cell types in each side of the brain are bilaterally symmetric, but for clarity different cells are shown in each half. Scale bar: 250 μm.

The broadly projecting morphological structure of VUM neurons, in conjunction with several physiological and molecular studies (Hammer, 1993; Schröter et al., 2007), makes VUM neurons likely candidates for representing sucrose via OA release. In the honey bee, at least two of these neurons—VUMmx1 and VUMmd1—each have a primary neurite that projects through the midline tract and gives rise to two symmetrical secondary axons that send collaterals to the antennal lobes, lateral horn (LH), lateral protocerebral lobe (LPL), and to the mushroom body (MB) calyces (Figure 1). In the fruit fly, the OA-VUMa2 neuron is similar to the honey bee VUMmx1 (Sinakevitch et al., 2005; Sinakevitch and Strausfeld, 2006; Busch et al., 2009). OA-VUMa2 sends secondary neurites to the posterior margins of the antennal lobes, where it projects fine ramifications into each glomerulus. OA-VUMa2 axons also follow the medial antenno-protocerbral tracts (m-APT) and connect the antennal lobe with the calyx and LH, while in the honey bee VUM axons follow the lateral antenno-protocerbral tracts (l-APT) and project to the LPL and MB calyx (Sinakevitch and Strausfeld, 2006; Busch et al., 2009).

In spite of the evidence that VUM neurons and OA are critical for appetitive learning in neural networks of the insect brain, very little is known about how OA receptors are integrated into those neural networks to drive these associations. We focus on the actions of VUM and OA on associative plasticity reported in the networks of the antennal lobe of honey bees and fruit flies (Fernandez et al., 2009; Kim et al., 2013). The honey bee antennal lobe is made up of approximately 160 glomeruli (Galizia et al., 1999; Robertson and Wanner, 2006), where axons from on average 400 olfactory receptor neurons (ORNs) converge onto 5–6 uniglomerular projection neurons (uPNs), assuming ~65,000 ORNs reported by Esslen and Kaissling (1976) and ~920 uPNs reported by Rybak (2012) (Figure 1) (Kelber et al., 2006; Kirschner et al., 2006; Nishino et al., 2009). In comparison, there are 56 glomeruli in the antennal lobe of adult fruit flies, which give rise to 150 uPNs (approximately 3/glomerulus) (Stocker, 2001; Laissue and Vosshall, 2008; Tanaka et al., 2012a). Glomeruli are functional coding units. In the fruit fly, as is also likely but not yet demonstrated in the honey bee, ORNs that converge to a glomerulus express the same receptors, which defines the range of odor ligands that activate the glomerulus (Laissue and Vosshall, 2008).

The structure of glomeruli is similar in both species. Each glomerulus has two distinct areas: the cortex, which contains ORN axon terminals, and the core, which lacks ORN arborizations (Fonta et al., 1993; Hummel and Zipursky, 2004; Tanaka et al., 2012a). In both species, PNs leave the antennal lobe through three main output pathways called the antennoprotocerebral tracts (APTs), named from Galizia and Rössler (2010) (Stocker et al., 1990; Fonta et al., 1993; Abel et al., 2001; Kirschner et al., 2006; Tanaka et al., 2008, 2012a). The l-APT connects the antennal lobe with LH, LPL and calyx of the MB. The m-APT connects the antennal lobe with the MB calyx, LPL and LH. And finally the medio-lateral APT (ml-APT) connects the antennal lobe with the LPL and LH. Three major subtracts of the ml-APT have been described in the honey bee (ml-APT1, ml-APT2, ml-APT3; Kirschner et al., 2006). Different types of local interneurons (LNs) interconnect glomeruli (Schafer and Bicker, 1986; Fonta et al., 1993; Olsen et al., 2007b; Shang et al., 2007; Seki et al., 2010; Meyer and Galizia, 2012; Girardin et al., 2013). GABAergic LNs are the largest group in both species (Schafer and Bicker, 1986; Ng et al., 2002; Okada et al., 2009; Seki et al., 2010). Two types of GABAergic LNs have been described based on different branching patterns in fruit fly antennal lobe glomeruli: LN1 (arborizations only in the core of the glomeruli) and LN2 (arborization in core and cortex regions)(Okada et al., 2009). In the honey bee, heterogeneous LNs are distinguished from homogeneous LNs by dense branching processes in one of the invaded glomeruli (Figure 1) (Fonta et al., 1993; Meyer and Galizia, 2012; Girardin et al., 2013). In addition, multiglomerular projection neurons (mPNs) connect the antennal lobe with the LPL and LH through the ml-APTs (Fonta et al., 1993; Abel et al., 2001; Tanaka et al., 2008; Okada et al., 2009; Seki et al., 2010).

In our previous study of the honey bee, we characterized antibodies against one type of OA receptor—AmOA1—and used them to demonstrate expression of AmOA1 in the inhibitory neurons of the antennal lobe and MB neuropil (Sinakevitch et al., 2011). In Sinakevitch et al. (2011), we also showed that anti-AmOA1 antibodies recognize the orthologous fruit fly OAMB receptor which is an important part of the reinforcement pathway for appetitive learning in the fruit fly (Han et al., 1998; Kim et al., 2013).

Here we extend our earlier study to describe in detail the morphology and neural circuitry of the antennal lobe. We show specifically how GABAergic processing in these networks is targeted by OA via AmOA1. The GABAergic system in the fruit fly has recently been extensively studied and described in detail elsewhere (Okada et al., 2009; Seki et al., 2010). We show strong similarities in expression of AmOA1 across the GABAergic targets in the antennal lobes of both species, which most likely reflects a conserved phylogenetic modulatory mechanism in olfactory networks. Finally, we use this information to propose a model for modulation of information processing in networks of the fruit fly and honey bee antennal lobes.

## Materials and methods

### Animals

Honey bees (*Apis mellifera*) were adult New World Carniolan pollen foragers from colonies maintained at Arizona State University. Fruit fly *(Drosophila melanogaster)* stocks and crosses were maintained at 22°C on a standard corn meal-yeast-agar medium supplemented with methyl-4-hydroxy-benzoate as a mold protector. The following strains were used: wild-type Oregon R; UASmcd8::GFP, used to express cell surface-associated GFP (Lee et al., 1999); GH146-GAL4, used as a marker of the projection neurons in antennal lobes of *Drosophila* (Stocker et al., 1997; Marin et al., 2002; Jefferis et al., 2007) and the APL neuron in the MB (Liu and Davis, 2009); and Or83b-GAL4, used as a marker of ORNs (Larsson et al., 2004). These strains were kindly provided by Dr. A. Fiala and Dr. T. Riemensperger (University of Wurzburg).

### Dye injection

Honey bee pollen foragers were collected at the entrance of the hive, briefly cooled, and restrained in individual harnesses. After recovering from cooling, honey bees were fed with 1 M sucrose solution and left undisturbed for 1–6 h before injection. Heads of the bees were fixed to the stage with soft dental wax (Kerr, Sybron Dental Specialties, Orange, CA, USA) in a way that allowed free movement of antennae and proboscis. A dissection knife was used to cut a window in the head capsule, dorsal to the joints of the antennae and rostral to the medial ocellus. The large pharyngeal glands were carefully moved until the MB vertical lobes [Strausfeld (2002) or alpha lobes in Rybak and Menzel (1993)] were visible, which are easily recognizable and serve as spatial reference for staining (Sachse and Galizia, 2002). The tip of a glass electrode coated with Rhodamine-dextran (Invitrogen, Grand Island, NY, USA) or with neurobiotin (Vector Laboratories, Burlingame, CA, USA), both prepared in 3% bovine serum albumin (BSA) solution (Sigma-Aldrich, St. Louis, MO, USA), was inserted into both sides of the protocerebrum rostro-lateral to the vertical lobes, aiming for both l-APT and m-APT, which contain the axons of uniglomerular (u)PNs (Abel et al., 2001). In order to reveal all APTs, the dye was deposited directly into the coarse area of the antennal lobe. The glass tip was held in this position until the dye bolus dissolved in the tissue (~3–5 s). The window was subsequently closed using the same piece of cuticle that was previously removed. Eicosane was used to glue and seal the cuticle. Immediately afterward, one of the antennae was cut transversally at approximately the middle of the scapus. A glass electrode coated with Rhodamine-dextran or neurobiotin (the respective tracer that was not used for the PNs in the same animal) was inserted into the antenna through the opened cavity and the electrode was rotated and moved until the coating was completely dissolved in the lumen of the antennae. The electrode was removed and the antenna was sealed with eicosane.

The next day, the piece of cuticle covering the brain was removed. Glands and trachea covering the brain were removed and the brain was rinsed with Ringer solution (130 mM NaCl, 6 mM KCl, 4 mM MgCl2, 5 mM CaCl2, 160 mM sucrose, 25 mM glucose, 10 mM HEPES, pH 6.7, 500 mOsmol; all chemicals from Sigma-Aldrich). For simultaneous staining with anti-synapsin or goat anti-AmOA1 antibodies, the brain was dissected and immediately fixed in 4% paraformaldehyde (Sigma-Aldrich) in phosphate buffer saline (PBS, pH 7.4) made from tablets (Sigma-Aldrich). For simultaneous staining with anti-GABA and goat anti-AmOA1 antibodies, the fixative was a mixture containing 1.5% glutaraldehyde [Electron Microscopy Sciences (EMS), Hatfield, PA, USA] and 2.5% paraformaldehyde (EMS) in 0.1 M sodium cacodylate buffer (EMS, pH 7.0), with 1% sodium metabisulfite (SMB, Sigma-Aldrich).

### Intracellular staining

For intracellular staining, the bee was mounted in the harness as described above and was alive during injections. Thin-walled borosilicate electrodes (resistance of 75–95 MΩ) with internal filament were used to stain one of the uPNs that was visualized by injection of Alexa-488-dextran 3000 (Invitrogen) into the m-APT as described above. Electrode tips were filled with a mixture of 7% neurobiotin and lysine fixable Rhodamine-dextran 3000 (Invitrogen) in 2 M potassium acetate (Vonhoff and Duch, 2010). To prevent dye dilution, an air bubble was left between the tip and the shaft. After intracellular penetration of the PN, the dye was injected iontophoretically by applying constant depolarizing current of 0.5 nA amplitude for 10–12 min. Subsequently, the electrode was removed and the brain was dissected out from the head capsule for fixation in 4% paraformaldehyde in PBS. Preparations were washed 6 × 30 min in PBS with 0.5% Triton X-100 (PBST), pH 7.4, then incubated with Streptavidin-Cy3 (Jackson ImmunoResearch Laboratories, West Grove, USA) to reveal neurobiotin in the cell.

### Immunocytochemistry

Mouse monoclonal anti-synapsin antibodies (SYNORF1; clone 3C11) were raised against bacterially expressed fruit fly synapsin. The anti-synapsin antibodies were kindly provided by E. Buchner, University of Wurzburg, Germany. These antibodies, which recognize presynaptic sites of neurons, were used here as a marker for synaptic neuropil in the antennal lobe of the honey bee. We employed the protocol originally used for studies of the honey bee brain with these antibodies (Brandt et al., 2005).

GABA antiserum (GEMAC, Talence, France) was raised in rabbits using GABA conjugated by glutaraldehyde to BSA, bovine hemoglobin, or poly-L-lysine. Antiserum specificity has been described elsewhere (Seguela et al., 1984; Sinakevitch et al., 1996, 2003, 2011; Strausfeld et al., 2003; Sinakevitch and Strausfeld, 2004). We used affinity purified goat anti-AmOA1 antibodies to describe the AmOA1 receptor distribution in the honey bee brain. Antibody specificity and staining controls in the bee brain were described in detail previously (Sinakevitch et al., 2011).

To immunostain the AmOA1 receptor ortholog in *Drosophila*, we used an AmOA1 antiserum from rabbit, which was previously used to study the distribution of AmOA1 receptors in the honey bee brain (Sinakevitch et al., 2011). This antiserum recognizes at least one isoform of the OA1 receptor in fruit fly: DmOA1A (CG3856-PB), the alternatively spliced isoform of the *Dmoa1* gene, which is identical to the OAMB (CG3856) receptor (Han et al., 1998; Balfanz et al., 2005; Maqueira et al., 2005). The specificity of these antibodies was demonstrated on oamb96 mutant flies, which lack part of the genomic region for oamb alleles (Lee et al., 2003), and is described in Sinakevitch et al. (2011, Figure S1).

#### Double-staining anti-synapsin and rhodamine-dextran (or neurobiotin) in the honey bee antennal lobe (n = 6, Table 1)

Following injection with Rhodamine-dextran (or neurobiotin) into the antenna, brains were fixed overnight at 4°C in 4% paraformaldehyde in PBS, then washed 6 × 20 min in PBST. After washing in PBST, brains were embedded in 8% agarose and 60 μm agarose sections were made using a Leica vibrating blade microtome VT1000S (Leica Biosystems, Germany). The sections were pre-incubated with 5% normal donkey serum (Jackson ImmunoResearch Laboratories) and the anti-synapsin antibodies were added to the sections in the dilution 1:10 and incubated overnight at room temperature. The sections were then incubated with PBST 6 × 20 min and secondary antibodies F(ab′)2 fragments of donkey anti-mouse IgG conjugated to Cy2 (Jackson ImmunoResearch Laboratories) were applied to reveal the synapsin staining (dilution 1:250).

**Table 1:** **Summary of all preparations used in this study**.

**Preparation labeled with antibodies to**						
**Injection site**	**Cell types revealed**	**Synapsin *n* = 12^c^**	**GABA *n* = 65^c^**	**AmOA1 and GABA^c^*n* = 26**	**AmOA1 *n* = 6^c^**	**Success rate^d^**
Antenna (Rhodamine or neurobiotin)	ORNs	*n* = 6, Figure 2A				100%
Rostral to MB vertical lobe	uPNs, sometimes VUM		*n* = 15, Figure 2C	*n* = 6, Figures 3A,B		90%
LPL	Mostly mPNs, sometimes a few uPNs^a^	*n* = 3	*n* = 9, Figures 5C,D,F	*n* = 3, Figure 5B		10%
LH and LPL	uPNs, mPNs		*n* = 19, Figure 2E	*n* = 3	*N* = 3^e^, Figure 2B insert, 3H	50%
Antennal lobe glomeruli	Local Neurons, uPNs, mPNs, ORNs		*n* = 10	*n* = 5, Figures 4D,E		90%
Antennal lobe aglomerular neuropil	all cell types and all tracts; m-APT, ml-APT, l-APT^b^		*n* = 12, Figure 2D	*n* = 9, Figures 4A–C,F		100%
Antenna (Rhodamine) +rostral to MB vertical lobe (neurobiotin)	mostly ORNs, mostly uPNs, sometimes VUM	*n* = 3			*n* = 3, Figures 3C–G	80%

a Injection site was not precise; occasionally the l-APT tract was included.

b These tracts were the primary focus for our study.

c Total number of preparations used for observations and conclusions in our study.

d Defined as getting fills in the targeted tracts.

e In these preparations, we made injection in the antenna to label ORNs.

In preparations, whenever neurobiotin tracer was used in the antenna, a 1:250 dilution of Streptavidin-Cy3 was added during the incubation of secondary antibodies. Then preparations were thoroughly washed in PBS and embedded in 80% glycerol.

#### Anti-GABA staining (n = 10)

Honey bee brains were removed in fixative containing 1.5% glutaraldehyde and 2.5% paraformaldehyde in 0.1 M sodium cacodylate buffer with 1% SMB. After fixation overnight at 4°C, whole brains were incubated for 15 min in 0.05 M Tris-HCl-SMB buffer pH 7.5 containing 0.5% NaBH4. After washing in 0.05 M Tris-HCl-SMB buffer, brains were embedded in 8% agarose and separate brains were cut into sections 35, 40, or 60 μm thick. After washing in 0.05 M Tris-HCl-SMB buffer with 0.5% of Triton X100 (TX), pH 7.5, sections were pre-incubated with 5% normal donkey serum for 1 h. Then anti-GABA antibodies were added to brain sections in a dilution 1:1000 in 0.05 M Tris-HCl-SMB-TX, for overnight incubation at room temperature. After washing in 0.05 M Tris-HCl-TX, pH 7.5, F(ab′)2 fragments of donkey anti-rabbit antibodies conjugated to either Cy3 or Cy5 (Jackson ImmunoResearch Laboratories, diluted 1:250 in 0.05 M Tris-HCl-TX) were used as secondary antibodies overnight at room temperature. After a final wash in 0.05 M Tris-HCl buffer pH 7.5, the sections were mounted on slides in 80% glycerol.

#### Anti-GABA staining after neurobiotin injection into the honey bee brain (n = 65, Table 1)

Following dye injections, brains were treated as described in the previous section. To reveal neurobiotin, after incubation with anti-GABA primary antibodies, Streptavidin-Cy2 (dilution 1:250, Jackson ImmunoResearch Laboratories) was added to the solution together with secondary antibodies conjugated to Cy3 or Cy5 as described above.

In control preparations, where anti-GABA was omitted, the secondary antibodies did not show any detectable staining. Sections of the honey bee brain without neurobiotin also did not show any detectable staining after incubation with both secondary antibodies and Streptavidin-Cy2 (data not shown).

#### Triple-staining with anti-AmOA1, anti-GABA and neurobiotin injected brains (n = 26, Table 1)

For simultaneous staining of AmOA1 and GABA in neurobiotin injected brains, brains were fixed and processed as described for anti-GABA staining. The brain sections were preincubated with 5% normal donkey serum for 1 h and, then simultaneously with both primary antibodies: goat anti-AmOA1 (1:1000) and rabbit anti-GABA (1:1000) overnight at room temperature. After a thorough wash in 0.05 M Tris-HCl-TX, secondary antibodies, F(ab′)2 fragments of donkey anti-goat IgG-Cy2 and F(ab′)2 fragments of donkey anti-rabbit IgG-Cy5 together with Streptavidin-Cy3 (all from Jackson ImmunoResearch Laboratories), were added overnight in dilution 1:200. The appropriate controls for goat anti-AmOA1 and anti-GABA stainings were described in detail previously (Sinakevitch et al., 2011). As controls for the specificity of the secondary antibodies, all secondaries were incubated with sections that had only one of the primary antibodies. The staining did not show any cross-reaction between the secondary antibodies and Streptavidin-Cy3. Streptavidin-Cy3 did not interact with any structure in the absence of the neurobiotin in the bee brain.

#### Triple staining with anti-AmOA1, neurobiotin and rhodamine-dextran injected brains (n = 6, Table 1)

After Rhodamine-dextran injection, brains were fixed in 4% paraformaldehyde in PBS overnight, then washed 6 × 20 min in PBST. After washing in PBST, brains were embedded in the 8% agarose and 60 μm brain sections were made. The sections were pre-incubated with 5% normal donkey serum for 1 h and then goat anti-AmOA1 antiserum (1:200) was added for incubation overnight at room temperature. The secondary antibodies were F(ab′)2 fragments of donkey anti-goat IgG conjugated to Cy2. Streptavidin-Cy5 (Jackson ImmunoResearch Laboratories) was used to reveal neurobiotin (dilution 1:250). Preparations were then thoroughly washed in PBS and embedded in 80% glycerol.

#### Anti-synapsin and anti-GFP staining in fruit flies (n = 5)

Whole heads of Or83b-GAL4; UAS-msd8-GFP were fixed in 4% paraformaldehyde in PBS. The eye and edges of the head capsules were cut off for rapid fixation of the brain. Preparations were embedded in 8% agarose and cut into 50 μm thick sections. Brain sections were washed 6 × 20 min in PBST, and preincubated for 1 h in 5% normal goat serum in PBST. The anti-synapsin (1:10) and anti-GFP chicken polyclonal antibodies (1:1000) (abcam, Cambridge, UK) were applied simultaneously to sections for overnight incubation at room temperature. The primary antibodies were diluted in PBST. After washing in PBST 6 × 1 h, secondary antibodies F(ab′)2 fragments of goat anti-mouse IgG conjugated to Cy5 (Jackson ImmunoResearch Laboratories) and Alexa 488 goat anti-chicken IgG (Invitrogen), diluted in PBST (1:250) were applied for 6 h at room temperature. After final washing in PBST 6 × 1 h, sections were mounted in 80% glycerol.

#### Anti-GABA and anti-AmoA1 in fruit flies (n = 10)

Whole heads were placed in fixative containing 2% glutaraldehyde in 0.1 M sodium cacodylate buffer with 1% SMB, pH 7.0. After fixation, semi-opened brains were incubated for 15 min in 0.05 M Tris-HCl-SMB buffer pH 7.5 containing 0.5% NaBH4 to saturate double bonds. After washing in 0.05 M Tris-HCl-SMB buffer, heads were embedded in 8% agarose and cut into 50 μm sections. To do the co-localization study of anti-AmOA1 with anti-GABA, both of which were raised in rabbit, we first did staining on consecutive sections: one section was labeled with anti-GABA and anti-GFP and the section next to it with anti-AmOA1 and anti-GFP. In order to obtain anti-GABA staining in one section and anti-AmOA1 in an adjacent section, we separated the sections of one brain in two wells of a 24-well nunc plate: odd sections in one well and even sections in a second well. After washing in 0.05 M Tris-HCl-SMB-TX, sections were preincubated with 5% normal swine serum (Dakopatts a/s, Glostrup, Denmark) for 1 h. “Odd” agarose brain sections were then incubated with anti-GABA antiserum (1:1000) and chicken polyclonal to GFP (1:1000), and “even” agarose brain sections were incubated with anti-AmOA1 and chicken polyclonal to GFP (1:1000) overnight. Primary antibodies were diluted in 0.05 M Tris-HCl-SMB-TX. After washing in 0.05 M Tris-HCl-TX, Alexa 488 goat anti-chicken IgG and goat anti-rabbit antibodies conjugated to Alexa 555 (Invitrogen) or Cy5 (Jackson ImmunoResearch Laboratories) diluted 1:250 in 0.05 M Tris-HCl-TX were used as the secondary antibody overnight. After a final wash in 0.05 M Tris-HCl, sections were embedded in 80% glycerol. The staining sections were compared and then AmOA1 staining sections were processed for anti-GABA staining. Sections labeled with anti-AmOA1 (and anti-GFP) were detached from slides, washed in PBS, and postfixed in 4% paraformaldehyde in PBS with 1% SMB for 20 min in order to deactivate antibodies of the first sequence of staining, and then processed for anti-GABA staining. We monitored two consecutive brain sections and used them as controls for double staining, where 0.05 M Tris-HCl buffer with 5% swine serum replaced GABA antiserum or AmOA1 antiserum. In these controls, we did not observe any interactions between antisera.

### Confocal microscopy

Digital images were captured with a Leica TCS SP2 or TCS SP5 confocal laser scanning microscope (Leica, Bensheim, Germany) using a Leica HCX PLAPO CS 40_ oil-immersion objective (numerical aperture: 1.25) or a Leica HCX PLAPO CS 100_ oil-immersion objective (numerical aperture 1.40) with appropriate laser and filter combinations. Stacks of optical sections at 1 μm spacing were processed using Leica software. Size, resolution, contrast, and brightness of final images were adjusted with Adobe Photoshop software. Images are either a single slice or flattened confocal stacks (maximum intensity projections). Images of *Drosophila* were collected on a Zeiss LSM 510 confocal microscope (Carl Zeiss, Oberkochen, Germany). Groups of four to ten serial 0.5-μ m optical sections (1,024 × 1024 at 8-bit color depth) were scanned using 40 × 1.0 or 63 × 1.4 oil iris Plan-Apochromat objectives. Images were stored as TIFF files and edited in Adobe Photoshop CS2.

#### Notes on immunostaining and injections (see Table 1 for a summary of the preparations used in this study)

Immunostaining for GABA and AmOA1 was best in the honey bee when animals were sacrificed immediately after collection. During optimization of the co-staining procedures by dye/or tracer with immunostaining for GABA and AmOA1, we found that it was crucial for animals to be alive and able to respond to stimuli (e.g., extension of the proboscis to food by honey bees) before being sacrificed. Nearly dead or dead animals nevertheless gave us good results for neuroanatomy of injected cells and processes, but immunostainings for GABA and AmOA1 were difficult to interpret, especially in glomeruli, for multiple reasons: background staining was too high (no differences in staining between cells and neuropil structures), staining was patchy and inconsistent, staining was absent, or the antennal lobe was obviously deformed. In Table 1, we summarized only preparations that had good quality immunostaining for neurobiotin injections.

In addition, staining for AmOA1 in particular was variable from animal to animal. This may reflect biologically meaningful variation in variables we did not control for in this study (e.g., age, caste, genotype, experience). However, we focus only on general patterns here.

Other difficulties that we found in our technique concern the way we deposited dye, especially in LPL, and LPL and LH; this gave us a very low yield of successful preparations even when the same group of neurons appeared in a sample. Different methods of injection should be used in the future to characterize mPNs in ml-APTs. For example, precise placement of injections with dye-filled microelectrodes in ml-APTs only, or use of mPNs markers (when available) would improve reliability of future results. Nevertheless, our preparations have been adequate for drawing conservative conclusions concerning GABAergic mPNs.

## Results

### Anatomy of glomeruli in the honey bee antennal lobe

#### Olfactory receptor neuron (ORN) terminals define the structure of the glomerular cortex

**Figure 2:**
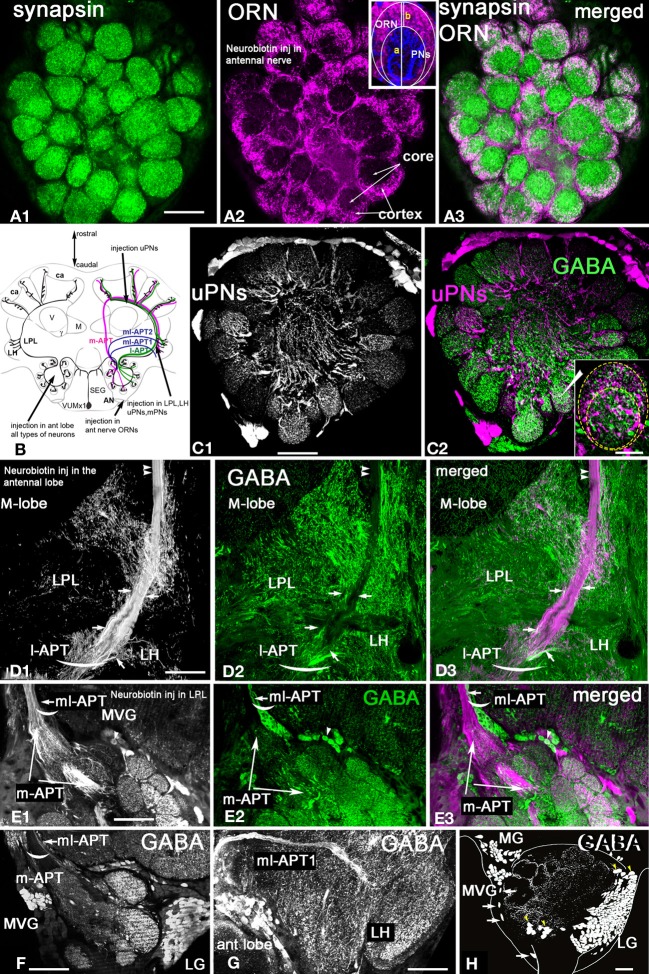
***Apis mellifera*.** General morphology of the antennal lobe and glomerulus. GABAergic neurons in the honeybee antennal lobe are local interneurons and multiglomerular PNs that branch into LPL and LP. **(A)** Frontal section of the honeybee antennal lobe immunostained with anti-synapsin antibodies (green) and Rhodamine-dextran labeled olfactory receptors neurons (magenta). **(A1)** Anti-synapsin (green) shapes the synaptic neuropil of the antennal lobe in all glomeruli, highlighting the potential synaptic connections between different types of neurons. **(A2)** Rhodamine-dextran injection into the antennal nerve revealed olfactory receptor neuron (ORN) endings (magenta) surrounding each glomerulus to form the cortex layer. The core area of glomerulus is free of ORNs. **(A3)** The merged images of the cortex area of the ORN endings in the antennal lobe overlapped with the area marked by anti-synapsin (white) indicate that ORNs synapse and receive synapses from other antennal lobe neurons in the cortex rind of glomerulus. Insert in **(A2)**: The schematic of the glomerulus overlaid on the middle section indicated by the image of the projection neuron dendrite (blue, PNs) and ORNs (magenta). The distribution of PN dendrites in the core and cortex of the glomerulus in the section made through the midline of the glomerulus demonstrates that large axons of PNs are in the core area and fine dendrites of PNs in the cortex where they overlap with ORNs: b-the length of the cortex area in the center, a-the length of the glomerulus. **(B)** Schematic view of the olfactory pathways in the brain of the honey bee where arrows show the site of Rhodamine-dextran or/and neurobiotin injections. The octopaminergic neuron VUMmx1 has a cell body in the subesophageal ganglion (SEG) and carries information along the olfactory pathway from the antennal lobe to the lateral horn (LH), lateral protocerebral lobe (LPL), and the MB calyx (ca). The tracts that carry the uniglomerular PNs are l-APT and m-APT, while the two tracts for multiglomerular mPNs are ml-APT 1,2. **(C)** Double stainings of uPNs (magenta) and anti-GABA (green) in the antennal lobe demonstrate that uniglomerular PNs are not GABAergic. **(C1)** Injection into the l-APT and m-APT as indicated in **(B)** revealed uPNs with dendrites in both core and cortex areas of glomeruli. **(C2)** Merged images of GABA (green) and uPNs (magenta) indicate that cell bodies of the uPNs are not GABAergic. White labeling in the glomerulus is due to overloaded dye in PNs and not co-localization, as illustrated in the image of the glomerulus with uPN dendrites and anti-GABA staining in the insert of **(C2)**. **(D)** Double labeling of the l-APT **(D1** single image) and anti-GABA (**D2** green single image) on the frontal section of the honey bee protocerebrum. The l-APT stained by injection in the antennal lobe **(D3)** The merged image illustrates that the two axons framing l-APT are GABAergic (white) and connect to the lateral protocerebrum (LH), another l-APT GABAergic fiber originating from antennal lobe branches in the lateral protocerbral lobe (LPL). Double arrows indicate the absence of anti-GABA in the uPN l-APT axons before their entry to the MB calyx. **(E)** Double staining of the m-APT and ml-APT revealed by injection of dye into LPL (single image **E1**, magenta in **E3**) and anti-GABA (green in **E1**and **E2**) **(E1)** uPNs in m-APT tract **(E2)** Anti-GABA staining manifests only in the section that we identify as the beginning of the ml-APT **(E3)** The m-APT is not stained with GABA, however a few GABAergic fibers are in the m-APT. These fibers are in the lateral part of the m-APT exiting from the antennal lobe that we identified as ml-APT 1,2(white merge image). **(F)** GABA staining in the m-APT and the two groups of GABAergic neurons in the frontal section of the antennal lobe are made in the area of the m-APT tract. The axons from the ml-APT are indicated by arrows. **(G)** GABA staining is in the ml-APT-1 that branches to the ventral part of the LH. **(H)** Schematic presentation of GABAergic cell clusters made after ten frontal brain sections (35 μm each) stained with GABA. The three groups of neurons identified are MVG, MG, and LG. Four neurons are identified as Giant MVG GABAergic neurons: they have a defined location and large somata (arrows). All figures show the right part of the brain: the middle of the brain is on the left and the lateral on the right. V, vertical lobe; γ, gamma lobe of MB. Scale bar: **A**, **C–H** = 35 μm; insert in **C2** = 15 μm.

Antibodies against synapsin label all presynaptic sites (Klagges et al., 1996), and here they revealed synaptic connectivity in all glomeruli of the antennal lobe (Figures 2A1,A3). In order to reveal ORN endings in the glomeruli, Rhodamine-dextran was deposited into the antennal nerve, as illustrated schematically in Figure 2B (see methods for details). The dye was taken up by ORN axons, which enter the antennal nerve through four tracts (T1–T4; Abel et al., 2001; Kirschner et al., 2006). Staining revealed axon endings in the cortex of each glomerulus (cortical layer; or glomeruli rind Figure 2A2) but not in the core. This pattern is characteristic of glomeruli in the T1-T3 tracts. In contrast, glomeruli innervated by T4 tracts receive ORNs in the entire glomerulus (not shown here; Arnold et al., 1985; Galizia et al., 1999; Nishino et al., 2009; Kreissl et al., 2010). We limit the description of glomerulus anatomy below to the T1 to T3 tracts.

ORNs are highly enriched with synapsin and they have targets in the cortex (Figures 2A2,A3). Glomeruli are roughly “egg-shaped” with the narrower end oriented toward the aglomerular area of the antennal lobe. uPNs enter the glomerulus through the aglomerular neuropil and have large fibers in the glomerulus core. In addition, they have fine branches extending into the cortex (Figure 2A2 insert) where they overlap and presumably make synaptic contact with ORN axons. The ORNs homogeneously innervate the glomerulus making up the lateral walls (approximately 5–10 μm thick) and a cap-like top (approximately 10–20 μm thick, the portion of the cortex indicated with b in Figure 2A2 insert). Sections where the glomerulus was cut through the midline (the absence of overlaying ORN fibers and presence of the large fibers of PNs in the core) were used to measure the length and width of core and cortex at the midline of the glomerulus. We estimate that, regardless of the size of the glomerulus, the ratio between the core and cortex areas measured at the midline of length was as follows: **b** (length of cortex in the midline)/**a** (length of glomerulus in the midline (core + cortex)) = 0.29 ± 0.05 μm [*SD*, *n* = 16 glomeruli that have ORN fibers in the cortex (Figure 2B insert)].

#### Structure of uniglomerular (u)PNs in the antennoprotocerebral tracts (APT)

In order to reveal uniglomerular PNs, neurobiotin tracer was injected into an area between the MB calyx and the vertical lobe (Figure 2B) where both tracts (l-APT and m-APT) meet to send their axons in the MB calyx. Each glomerulus is innervated by an estimated 5–6 uPNs (Rybak, 2012), and, in contrast to the ORNs, the dendrites of these uPNs cover the core and cortical layer of each glomerulus homogeneously (Figure 2C1). The uPNs enter glomeruli from the aglomerular neuropil and form large branches (2–4 μm) at the beginning of the core area. Fine arborizations of the uPN dendrites (thickness ranges between 0.2–0.7 μm) are tightly packed in the cortex. Because they overlap with ORNs in the cortex, the fine arborizations of uPNs might be postsynaptic to the ORNs; however, direct synapses between ORNs and uPNs have not been conclusively shown.

#### uPNs of the m- and l-APT are not GABAergic

Co-staining of neurobiotin injected uPNs with anti-GABA antibodies revealed that uPNs do not exhibit GABA immunoreactivity in their cell bodies, dendrites, (Figure 2C2), or axons in l-APT (Figures 2D1–D3) and m-APT (Figures 2E1–E3, 4C–E). The white color in a few glomeruli in Figure 2C2 is due to close proximity of tightly packed uPN dendrites and GABAergic arborizations in low magnification. Higher magnification of a glomerulus, co-labeled with anti-GABA and neurobiotin injected uPNs, illustrates clearly that there is no co-localization of GABA within the dendrites of the uPNs in the glomerulus (insert in Figure 2C2). GABA immunoreactivity is distributed throughout the whole glomerulus in both cortex and core area, clearly originating from different GABAergic cells.

#### Approximately 17 GABAergic mPNs from the ml-APT branch in the LPL

In other preparations, neurobiotin was injected into the LPL and LH, which fills neurons that belong to the l-APT, m-APT, and ml-APT (Kirschner et al., 2006). The m-APT and ml-APT leave the antennal lobe together; Figure 2E1 shows a horizontal section at the beginning of both tracts as they exit the antennal lobe. Most uPN axons in the tracts from both m-APT and ml-APT neurons are labeled by neurobiotin (Figure 2E1). Anti-GABA antibodies stained groups of axons in the ml-APT tract (short arrow in Figure 2E2). Merging the images of neurobiotin injected neurons (magenta) and anti-GABA stained neurons (green) reveals that this group of GABAergic axons runs laterally at the beginning of the m-APT before turning into ml-APT tracts [matching arrow positions in Figures 2E2,E3, (white)]. The same GABAergic axons leaving the antennal lobe are illustrated in the frontal section of the antennal lobe (Figure 2F). These GABA stained axons leave the antennal lobe through the ml-APT1,2 tracts, and they then enter and branch into different areas of the LPL (Figure 2G). When we counted the number of these fibers in the frontal and horizontal sections of eight bee brains, we found that approximately 17 ± 3 GABA-positive fibers were in the ml-APT 1, 2.12 ± 2 in the ml-APT 1 and 5 ± 2 in the ml-APT-2 (ml-APT-2 is not illustrated here). We identified these neurons as mPNs according to Kirschner et al. (2006).

#### Most GABAergic neurons in the antennal lobe are local interneurons

Figure 2H shows the schematic reconstruction of the antennal lobe from the frontal sections stained with GABA antiserum. We identified three distinct soma groups of GABAergic neurons in the antennal lobe: the medio-ventral group MVG (Figures 2E,H), the medial group MG (Figure 2H) and the lateral group (LG). Most of the GABAergic neurons in these groups are LNs that interconnect glomeruli and do not exit the antennal lobe neuropil. The exceptions include approximately 17 GABAergic mPNs that leave the antennal lobe through the ml-APT, and at least four unidentified neurons that leave through the l-APT. In addition, two GABAergic neurons located in the most dorsal part of the medial group (MG in Figure 2H) leave the antennal lobe via midline bundles (these neurons are not illustrated here).

#### There are approximately 375 GABAergic local interneurons in the antennal lobe

We counted the cells in the frontal sections of three honey bee brains stained with anti-GABA antibodies. In total there are 402 ± 30 cells distributed as follows: MG (*n* = 30 ± 5) and MVG (*n* = 18 ± 5) and LG (*n* =350 ± 25). In addition, there are four giant medio-ventral GABAergic neurons with large somata, in the medial ventral part of the antennal lobe (arrows in Figure 2H). Because the injection of neurobiotin into the antennal lobe revealed both neurobiotin tracer and GABA within the same cell population, we can associate all of these GABAergic neurons with the antennal lobe (not illustrated here). The GABAergic cells located dorsally to the antennal lobe are not counted in our preparations because they are not co-localized with staining of the tracer injected into aglomerular area of the antennal lobe. The LG is the largest group of GABAergic neurons and covers the lateral area of the antennal lobe. Most of the LG neurons have a cell body size ranging from 8–10 μm. The exceptions are the larger GABA neurons (15 μm) that cluster on the top of the antennal lobe and the rostral part of the LG (yellow arrowheads in Figure 2H). Furthermore, as described by Kreissl et al. (2010), these neurons exhibit both alatostatin and GABA (*n* = 20, size = 15 μm) in their cell bodies, and they branch within the core and inner cortex area of each glomerulus.

#### Anti-AmoA1 antibodies label gabaergic processes in the glomeruli

**Figure 3:**
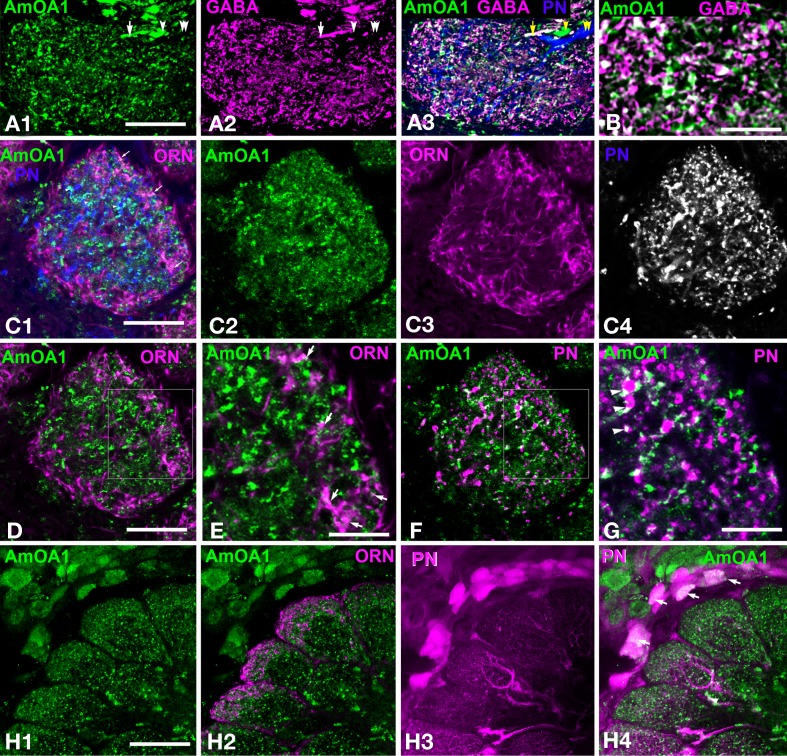
***Apis mellifera*: The GABAergic processes in glomeruli express AmOA1 (A) Triple labeling of the anti-AmOA1 (green A1) and anti–GABA (magenta A2) in a glomerulus with neurobiotin-injected uPNs (blue A3). (A3)** The fibers that enter into the glomerulus are GABAergic co-stained with AmOA1 (white, arrow), non-GABAergic processes co-stained with AmOA1 (green, arrowhead) and uPNs that do not co-localize with AmOA1 (blue, double arrowheads). **(B)** GABA and AmOA1 staining at higher magnification **(C)** Triple labeling of a glomerulus **(C1)** in which anti-AmOA1 (green, **C1** and **C2** single image) and ORNs were labeled by anterograde staining with Rhodamine-dextran (magenta **C1**, single image **C3**), and uPNs are shown by retrograde staining (blue **C1**, and black and white in a single image **C4**). **(D)** The same glomerulus as in **(C1)** but only ORNs (magenta) and AmOA1(green) are shown **(E)** illustrates a high magnification of the area shown in the square designating in **(D)**. Arrows show the close proximity of the AmOA1 stained profiles and ORNs. **(F)** The same glomerulus as in **(C1)** but only staining of AmOA1 and uPNs are shown. **(G)** A high magnification of the area shown by the square designated in **(F)**. Arrowheads show AmOA1 in the area that surrounds the uPNs fibers, which might be presynaptic to uPNs. **(H)** Detail of the antennal lobe labeled with anti-AmOA1 (green, **H1**) and anterogradely labeled ORNs (magenta **H2** in merge image). ORNs do not show the co-expression with AmOA1. **(H3)** PNs are labeled by injection into the LPL where three glomeruli from the lateral part of the antennal lobe are shown with cell bodies surrounding the antennal lobe. **(H4)** Merged image where anti-AmOA1 (green) and PNs (magenta) illustrate that there are possible co-localizations of AmOA1 in the cell bodies of subsets of PNs. Scale bars: **A,C,D,F** = 15 μm; **B,E,G** = 5 μm; **H** = 35 μm.

To examine AmOA1 staining in glomeruli, we co-labeled frontal sections of the antennal lobe with anti-AmOA1 antibodies and GABA antiserum and analyzed the distribution of staining within glomeruli (Figure 3A). AmOA1 immunostaining was variable across glomeruli by amount and intensity in the core and cortex area (compare Figures 3A1,C2,H1, from three different honey bee brains). An example of an antennal lobe glomerulus that exhibits a high level of anti-AmOA1 staining in the core and cortex area is shown in Figure 3A1. Furthermore, AmOA1 staining is expressed in both GABAergic (arrow) and non-GABAergic processes (arrowhead) but not in uPNs (double arrowheads in Figures 3A1–A3). GABA and AmOA1 immunoreactivities are also distributed throughout the whole area of the glomerulus (Figures 3A1,A2).

#### Anti-AmoA1 is not in the ORN endings and uPN dendrites

In order to study AmOA1 distribution on ORNs and uPNs, we injected Rodamine-dextran into the antennal nerve and neurobiotin in the m- and l-APTs into the area between the MB lobes (Figure 2B, Table 1). Then we applied anti-AmOA1 antibodies to frontal sections of the antennal lobe. All three stainings are illustrated in Figure 3C1 where the anti-AmOA1 staining is labeled in green (Figure 3C2), the ORN endings in magenta (Figure 3C3) and uPN dendrites in blue (Figure 3C4). Most colors are not mixed, which indicates that most of the AmOA1 staining is not in uPNs or ORNs (Figures 3C1,D–G). There are only a few mixed colors: yellow for ORNs and anti-AmOA1 (arrows); light green showing possible co-staining of uPNs and anti-AmOA1 arrowheads in Figure 3C1. Higher magnification revealed that white colors could indicate a possible co-staining of anti-AmOA1 and ORNs (arrows in Figure 3E) and uPNs (arrowheads in Figure 3G), or more likely it could indicate close proximity of ORN and anti-AmOA1 stained processes.

Data from our light microscope analysis showed clear differences between the distribution of AmOA1 receptor in the GABAergic processes and in the uPNs and ORNs. We found that anti-AmOA1 antibodies are also expressed on the cell body of the PNs in the medio-ventral group (Figures 3H1–H3), and may co-stain their axons in the glomeruli (Figure 3H4). These neurons might belong to mPNs that leave the antennal lobe via ml-APT1,2.

#### Anti-AmoA1 labels gabaergic processes and some kenyon cells but not axons or endings of uPNs in calyces and LPL

**Figure 4:**
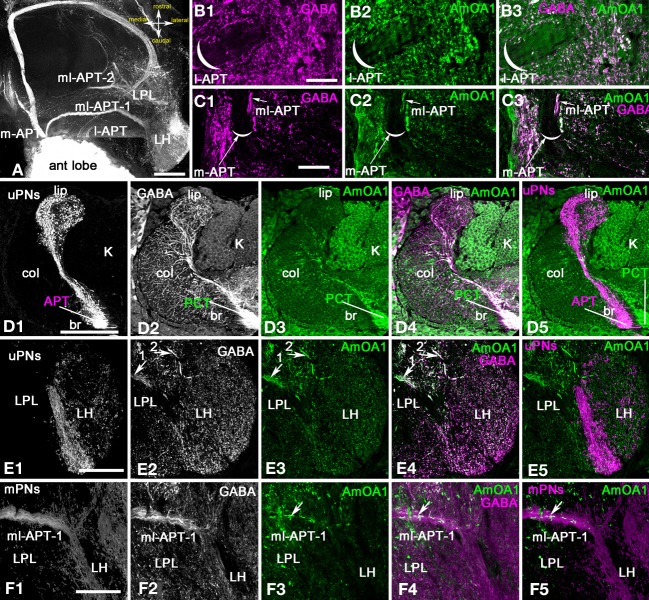
***Apis mellifera*: The AmOA1 immunostainings in the central brain neuropils (LPL, PL, and MB calyx) are co-localized in the GABAergic neurons but not in uPNs. (A)** An injection of a neurobiotin tracer into the antennal lobe revealed all antenna-protocerebral tracts (APTs). **(B)** The origin of l-APT tracts did not show GABA (magenta, **B1**) and AmOA1 (green, **B2**) staining inside of the tract. **(B3)** The merged image shows white fibers indicating the co-localization of anti-GABA and Anti-AmOA1 in the area surrounded the l-APT. **(C)** The beginning of the m-APT in a frontal section of the brain in which the anti-GABA (**C1**, magenta) and anti-AmOA1 (**C2**, green) are co-localized in the lateral part of the tract (white image merge in **C3**). The medial part of the m-APT containing axons from uPNs is not GABAergic. **(D)** The calyx of the MB on the frontal section of the brain with injected subsets of uPNs from l-APT and m-APT (**D1**, single image) stained with anti-GABA (**D2**, single image), and anti-AmOA1 (**D3**, single image). **(D1)** uPNs ending in the lip (lip) and basal ring (br) of the MB calyx. uPNs enter to the calyx via APT. **(D2)** Anti-GABA profiles originating from PCT (feedback) neurons that enter to the calyx via PCT (protocerebral tract) can be found in all calyxes. **(D3)** Anti-AmOA1 immunostained the Kenyon cell (K) as well as a subset of the PCT neuron GABAergic endings as demonstrated in the merged image **(D4)** (anti-GABA magenta, and anti-AmOA1 green). **(D4)** The white area in the merged image clearly indicates the distribution of anti-AmOA1 staining within the subset of the GABAergic endings. **(D5)** The merged image of the uPNs (magenta) and anti-AmOA1 do not show co-localization of AmOA1 within uPNs ending in the calyx. **(E)** The Lateral Protocerebral Lobe (LPL) with triple staining injected m-APT uPNs dendrites **(E1)**, anti-GABA **(E2),** and anti-AmOA1 **(E3)**. **(E4)** Merged images of anti-GABA (magenta) and anti-AmOA1 (green) revealed co-staining in the fibers from ml-APT 1, 2 (arrows) and in the GABAergic processes of the LPL. **(E5)** There is no evidence for co-localization in merged image of uPNs (magenta) and anti-AmOA1 (green). **(F)** The anti-AmOA1 stainings only the subset of the GABAergic mPNs in the ml-APT1. **(F1)** The neurobiotin deposits in the antennal lobe revealed an ml-APT tract that we associated with mPNs. **(F2)** GABA immunoreactivity in the ml-APT-1. **(F3)** Anti-AmOA1staining in the ml-APT1. **(F4)** As shown in merged staining (white) only a few GABAergic fibers (magenta) are co-stained with anti-AmOA1 (green). **(F5)** The same for the merged image showing injected mPNs fibers (magenta) and anti-AmOA1 (green). Scale bar: **A,D–F** = 100 μm; **B,C** = 20 μm.

Anti-AmOA1 did not label axons of uPNs in the m-APT and l-APT (Figures 4A,B2,C2). However, it labeled GABAergic processes that frame the l-APT (Figures 4B1,B3), and it also labeled the subsets of GABAergic mPN axons of the ml-APT 1,2 (Figure 4C1). In both tracts, only the GABA-positive processes contain AmOA1 receptors, meaning that the axons of the uPNs do not express AmOA1. Figure 4D1 illustrates terminals of the uPNs in the basal ring and lip of the calyx of the MB. This same area receives GABAergic innervation from GABAergic protocerbral tract neurons (PCT; Figures 1, 4D2) (Sinakevitch et al., 2011). The anti-AmOA1 is mostly co-localized with the GABAergic endings of the PCT neurons in the calyx of the MB [Figure 4D4, white processes on merged image of anti-GABA (magenta) and anti-AmOA1 (green)]. Anti-AmOA1 also labeled many cell bodies of the Kenyon cells (Figures 4D3,D4). Anti-AmOA1 is not exhibited on the uPNs endings in the MB calyx (Figure 4D5). Further, the GABA processes co-localize with anti-AmOA1 but not with uPN outputs in the basal ring and lip of the calyx.

The uPNs also branch in the LH, which is in the latero-ventral area of the protocerebrum (Abel et al., 2001; Kirschner et al., 2006). The uPNs from the m-APT and l-APT tracts branch in different parts of the LPL. The m-APT uPNs end in the ventral area of the LH, while the l-APT ends in the dorsal part of LH (Kirschner et al., 2006).

Figure 4E1 shows the endings of the m-APT in the LH, and these endings are not co-labeled with anti-AmOA1 or with anti-GABA. The AmOA1 immunoreactivity is localized in GABAergic terminals. Both the ml-APT-1 and ml-APT-2 tracts ended in the LPL in different areas; ml-APT-1 ended in the most ventral section of the LPL and ml-APT-2 in the dorsal section. Anti-AmOA1 is stained in the axons and endings of the GABAergic ml-APT-1,2 mPNs (Figures 4E,F).

#### Distribution of GABAergic mPNs in antennal lobe glomeruli

**Figure 5:**
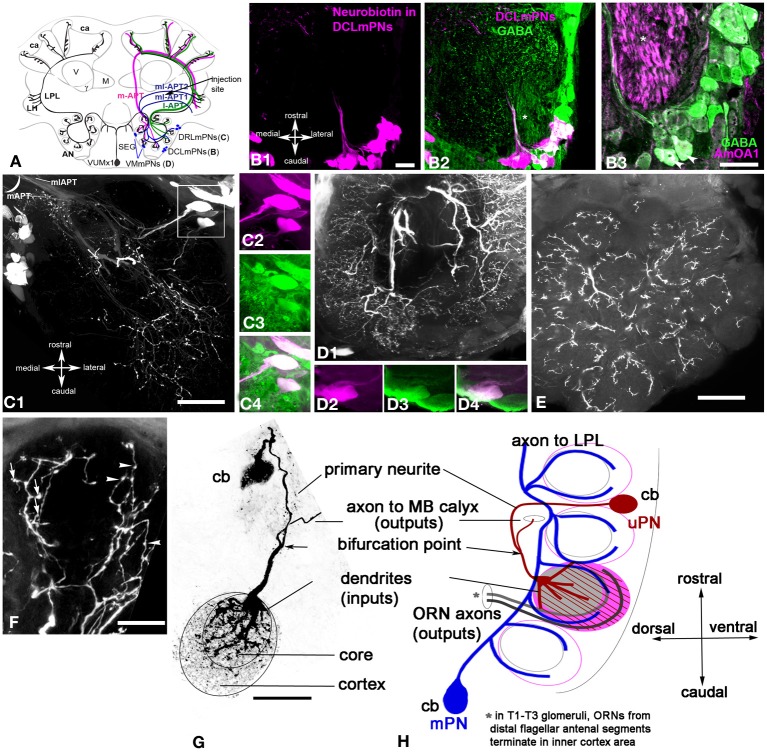
***Apis mellifera.*** The mPNs branching in the glomeruli as revealed by neurobiotin injection into the lateral protocerebral lobe. **(A)** Schematic of the honey bee brain that illustrates octopaminergic neurons that branch in the antennal lobe, lateral protocerebral lobe (LPL) and calyx of the MB. Neurobiotin injected into the LPL revealed three groups of neurons named as follow DCLmPNs **(B1)**, DRLmPNs **(C1)**, and VMmPNs **(D1)**. A subpopulation of the neurobiotin injected neurons from DCLmPNs (magenta **B1** and **B2**) were co-labeled with GABA (green). **(B3)** A higher magnification of the GABA neurons co-stained with anti-AmOA1: arrowheads indicate the GABAergic DCLmPNs neurons that co-labeled with anti-AmOA1. **(C1)** There are three neurons in the DRLmPNs group that are located dorsally in a lateral cluster of the most caudal part of the antennal lobe. These neurobiotin labeled neurons (**C2**, magenta) are co-stained with anti-GABA (**C3**, green single image). **(C4)** The merged image shows the anti-GABA (green) and neurobiotin (magenta) labeled neurons. **(D)** An image from the brain section of the antennal lobe where few axons from different cells connect different glomeruli. One neuron we identify from VMmPNs group, it was possible to follow the neurite from cell body and its branching into glomeruli. This neuron (**D2**, magenta single image) co-localized with GABA (**D3**, green single image) is shown in the merged image in **(D4)**. **(E)** The section from the same preparation through most ventral part of the antennal lobe. The branching pattern of the neurons in the glomeruli is in the cortex area. **(F)** The mPNs ending in the glomeruli cortex revealed spine like (arrows) and bleb-like structures (arrowheads). **(G)** The anatomy of single uPN revealed by intracellular injection into the cell body on the left, collapse frontal view. **(H)** The right schematic demonstrates the branching pattern of the uPN and mPN in the antennal glomeruli. uPN has the thick fibers in the core and fine arborization in the cortex (red), mPNs fibers are in outer area of the core and in the inner cortex. Asterisks in **(B2,B3)** indicated the axons from antennal nerve traveled to mechanosensory and motor center neuropil. Scale bar: **B** = 20 μm, **C1,D2,E** = 50 μm, **F,G** = 15 μm.

To identify how mPNs branch in the antennal lobe glomeruli, we injected neurobiotin into multiple sites close to the LH and rostral to the MB vertical lobe with subsequent anti-GABA and anti-AmOA1 staining (Figure 5A, LPL and LH sections in Table 1). We acknowledge that this method has its limitations. Not all the mPNs in the ml-APT could be revealed and sometimes a few uPNs were also stained (especially from the injection site in the LPL). However, by examining the neurobiotin tracer staining in six preparations with similar groups of neurons filled from 15 total preparations (Table 1) through the LPL as indicated in Figure 5A, we found three groups of cell bodies that co-labeled with GABA. The largest group is Dorso-Caudal Lateral mPNs (DCLmPNs, Figures 5A,B). We also identified at least two GABAergic neurons in the Dorso-Rostral Lateral mPNs group (DRLmPNs, Figures 5A,C). Finally, we identified at least three GABAergic neurons in the Ventro-Medial mPN group (VMmPNs, Figure 5D). The somata described here belong to the ml-APT because it was possible to follow their axons from the beginning of ml-APT to their cell body in these preparations. The methods that we used here gave us the approximate position and branching patterns of some of the GABAergic mPNs, however the numbers of the GABAergic fibers in the ml-APT tracts suggest that there might be many more with such patterns. It is important to note that ml-APT-1,2 also had neurobiotin labeled fibers that were not co-localized with anti-GABA staining.

The largest group of neurons that were filled with neurobiotin after LPL injection is located caudally at the most lateral part of the antennal lobe near the mechanosensory and motor center neuropil, where the antennal lobe connects to subesophageal ganglion (DCLmPNs, Figures 5B,C). In LPL-injected preparations, the number of neurons in this group varied from 30 to 40 due to the injection site. Not all the neurons from these groups exhibit GABA-like immunoreactivity (Figures 5B1,B2). We found that 15 ± 4 neurons (*n* = 6) in this group co-localized with GABA (Figure 5B2). Moreover, among those GABAergic neurons only two were co-stained by anti-AmOA1 in the cell bodies and primary axons (Figure 5B3). This is illustrated in higher magnification of the merged images of anti-GABA (green) and anti-AmOA1 (magenta) in Figure 5B3. Significantly, this is consistent with the data shown in Figures 4E,F, where only a few fibers from ml-APT1 that enter into the LPL are labeled with AmOA1. We could not identify the glomerular distribution of the dendrites from these neurons; however some of them might have arborizations in many glomeruli as shown in Figure 5D1. Note that axons from the antennal nerve to mechanosensory and motor center neuropils are stained with anti-AmOA1 (Figure 5B3). The following group, which we identify as DRLmPNs, is illustrated in Figure 5C, which demonstrates that the cell bodies lay laterally and rostrally in the relationship to the DCLmPNs group. All three neurobiotin filled cells shown in close up [Figure 5C2 (magenta)] co-localized with anti-GABA antibodies [Figure 5C3 (green, single image of GABA staining) and Figure 5C4 (merged image of neurobiotin and anti-GABA)]. The VMmPNs group reveals scattered cell bodies in the medial part of the antennal lobe; two cell bodies on the ventro-medial part of the glomeruli are illustrated in Figure 5D1. In higher magnification, both cell bodies are co-localized with anti-GABA staining [white merged images in Figures 5D4,D3 (anti-GABA, green), **5D2** (neurobiotin, magenta)].

We noted that a few thick fibers run through the coarse area of the antennal lobe extending branches to all glomeruli, however, due to an extensive branching pattern, it was difficult to identify how many fibers belong to one cell in the frontal sections of the brain. In the frontal section through the top of the antennal lobe, the branching in the glomeruli are seen mostly in the cortex area (Figure 5E). A higher magnification of a glomerulus revealed different structural patterns (i.e., bleb-like and spine-like endings) in the cortex area of the glomerulus (Figure 5F).

#### Dendrites of the uPNs and mPNs reveal differences in their connections to the core and cortex area of the glomerulus

Figure 5G illustrates the projected view of an image stack of a uPN labeled by intracellular neurobiotin injections. The cell body (15 μm) is on the surface of the antennal lobe between glomeruli, as shown in the schematic in Figure 5H. The primary neurites plunge vertically between glomeruli and run approximately 60 μm to bifurcating points toward either the MB calyces and to the glomerulus. A neurite (20 μm length from bifurcation point) runs through the coarse area of the antennal lobe close to enter a glomerulus through the core (Figures 5G,H). There the neurite gives rise to thick fibers that branch mostly in the core of glomerulus as well as fine, densely packed fibers that cover the entire glomerulus (see Figures 2C1, 5G,H). The area of the cortex of the glomerulus is connected with fine fibers of the uPN (Figures 5G,H).

The glomerular branching of the uPNs and mPNs is schematically drawn in the sagittal view of the antennal lobe (Figure 5H). In contrast to the uPNs that have branches all over the glomerulus, with thick fibers in the core and fine dendrites in the cortex, the mPNs branch only in the cortex. According to Nishino et al. (2009), the fibers of the ORNs are topographically organized within the glomerulus, where the ORNs from the most distal part of the antennal segment enter close to the core or inner area of the cortex and the ORNs from the proximal part of the antennal segment enter to the peripheral area of the cortex. There each glomerulus has two areas: the cortex, which receives ORNs, and the core, where ORN endings are not present. Both uPNs and mPNs are present in the cortex of the glomerulus and might receive excitatory input from the organized ORNs in this region (Figure 5H).

### Anatomy of glomeruli in the fruit fly antennal lobe

#### Olfactory receptor neuron (ORN) terminals also define the structure of the glomerular cortex in the fruit fly

**Figure 6:**
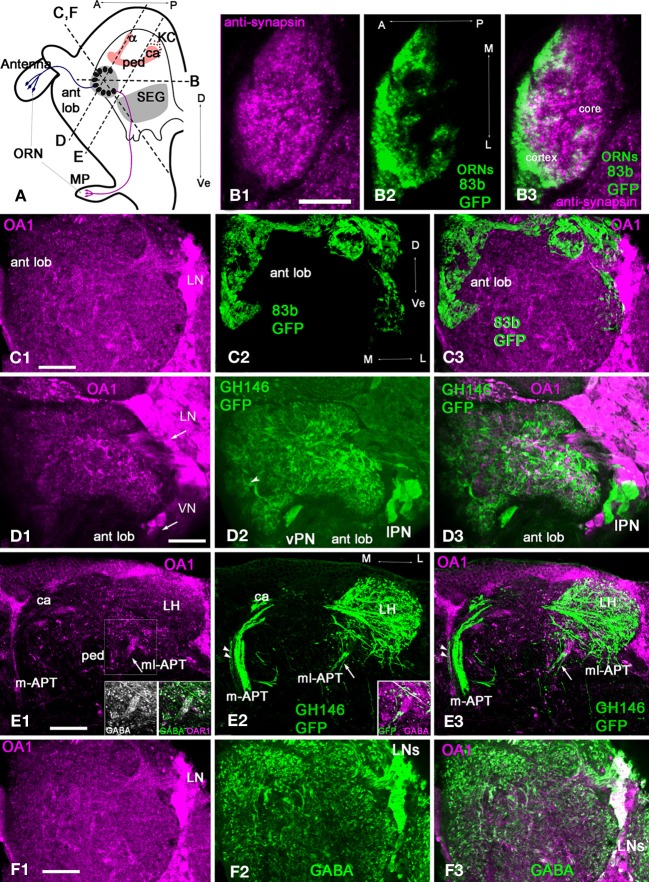
***Drosophila melanogaster*: a subpopulation of GABAergic neurons are co-stained with anti-OA1 antibodies. (A)** Schematic of the organization of the *Drosophila* olfactory system in sagittal view. MP, maxillary palp; ORN, olfactory receptor neuron; SEG, subesophageal ganglion; ant lob, antennal lobe; ca, calyx, ped, pedunculus; v, vertical lobe of MB; A, anterior; P, posterior; Dorsal, Ve- ventral. Broken lines indicate the approximate orientation of sections through antennal lobe (in **C,F,D**) and lateral protocerbrum **(E)**. **(B)** Section through the center of one glomerulus labeled with anti-synapsin (green, **B1**) and anti-GFP in ORNs (magenta, **B2**) in OR83b-GAL4;UAS-mcd8-GFP flies. The glomerulus has a core area where the ORNs do not branch **(B3)**. **(C1)** Anti-OA1 stained groups of cells and processes in the glomeruli and the aglomerular neuropile area of the antennal lobe in OR83b-GAL4;UAS-mcd8-GFP (**C1**, magenta) on the oblique frontal brain cross-section of the antennal lobe. **(C2)** In the same section, GFP (green) takes up a large percentage, or perhaps all, of the olfactory receptor endings in the antennal lobe glomeruli. **(C3)** The majority of the sensory neurons terminals do not label with the OA1 antiserum (magenta) with a few exceptions. **(D,E)** Anti-OA1 antibodies do not label the majority of uniglomerular projection neurons (uPNs). **(D1)** Here, anti-OA1 labeled clusters of cells surrounding the antennal lobes. These neurons are not projection neurons. GH146-GAL4; UAS-mcd8-GFP projection neurons expressed GFP (green) **(D2)**. In GFP expressing neurons there is no OA1 immunoreactivity as shown in our merged image **(D3)**, co-localization would show as white. **(E)** Anti-OA1 staining is absent in most uPN axons that leave the antennal lobe via the m-APT and branch in the calyx (ca) of the MB and lateral horn (LH); one exception is the axon shown by two arrowheads **(E1)**. mPNs leave the antennal lobe via the ml-APT, a large portion of the ml-APT fibers are OA1 positive (arrow) in **(E1,E3)**. These fibers also exhibit anti-GABA staining (inserts in **E1**). Three neurons that have their axons in ml-APT are also labeled with anti-GFP and GABA in GH146-GAL4; UAS-mcd8-GFP (**E2** and insert in **E2**). These neurons are not labeled with anti-OA1 in this brain preparation **(E3)**. **(F1)** In the same section as shown in **(C)** anti-OA1 (magenta) labels the cell bodies of laterally located neurons. The scattered, stained processes are in all glomeruli. **(F2)** The same sections labeled with anti-GABA antibodies; and GABA-like immunoreactivity is found in neurons with cell bodies in lateral and dorso-lateral clusters. These neurons supply GABAergic processes to the glomeruli. The merged image **(F3)** of the same sections shows the group of GABAergic neurons co-stained with anti-OA1 (white). Anti-OA1 staining is found in the cell bodies and processes in the glomeruli and in the aglomerular area of the antennal lobe. Arrows in **(C1)** and **(D1,F1)** indicate lateral neurons cluster (LN) and ventral cluster (VN) of anti-AmOA1 positives neurons. In **(D2)**, the arrowhead indicates thick fibers of the PNs entering the glomerulus that might correspond to the core area of the glomerulus. Scale bar: **A** = 10 μm, **B–F** = 20 μm.

We used the enhancer trap lines OR83b-GAL4× UASmcd8GFP, which express the GFP in ORNs, to reveal receptor neuron axon endings in the antennal lobe (Larsson et al., 2004). In this line, ORNs from the antenna and the maxillary palp (MP) have axons that terminate in the glomeruli (Figure 6A). ORNs from the antenna enter the antennal lobe from lateral and anterior positions, and ORNs from the MP travel dorsally through the suboesophageal ganglion (SEG) to enter the antennal lobe from a ventral posterior position (Figure 6A; Jefferis et al., 2004; Sweeney et al., 2007).

In the fruit fly, glomeruli have an outer cortex innervated by ORN terminals and a core area that lacks ORN terminals (Figure 6B). The glomerular structure in the fruit fly is similar to that of the honey bee. In horizontal sections (Figure 6A, section panel **B**), anti-synapsin antibodies labeled the entire glomerulus (Figure 6B1) and anti-GFP labeled the ORNs glomerulus (Figure 6B2). Like in the honey bee, ORNs terminate in the cortex (Figure 6B2). However, glomeruli in fruit flies do not exhibit strictly defined borders between the core and cortex, which makes estimation of the relative areas of core and cortex difficult. Finally, as in honey bees, the fruit fly antennal lobe contains glomerular and aglomerular neuropils (Figures 6C,D,F).

#### Most of the ORNs in fruit flies do not label with AmoA1

In general, anti-AmOA1 antibodies label the clusters of cells that surround the antennal lobe and are located dorso-laterally, laterally, and ventrally to the antennal lobe (Figures 6C1,D1,F1). We did not find co-localization of AmOA1 within the most ORNs terminals (Figures 6C1–C3).

#### Most uPNs in fruit flies do not label with anti-AmOA1

To study the distribution of the OA1 receptors in a uPNs we used the GAL4-GH146 line crossed with UAS –mCD8-GFP, which drives GFP expression in a large subset of uPNs and a few mPNs (Figures 6D,E) (Marin et al., 2002). We could then visualize via GFP the cell bodies and dendrites of uPNs. The uPNs are cholinergic (Stocker et al., 1997; Jefferis et al., 2002; Python and Stocker, 2002; Olsen et al., 2007a). Thick fibers from uPNs are in areas that might correspond to the core (Figure 6D, arrowhead), while fine ramifications of the PNs are all over the glomerulus. Most of the GFP labeled cell bodies and axons, as well as their endings in the MB calyx and LH, do not label with anti-AmOA1 antibodies (Figures 6D,E). Anti-OA1 staining is also absent in m-APT projection neurons and their outputs in the calyx (Figure 6E1), with the exception of one or two axons that co-localize with anti-OA1 in the medio-lateral part of the m-APT (Figure 6E2, two arrowheads in m-APT).

Most of the AmOA1 stained axons that project from the antennal lobe belong to mPNs in the ml-APT (Figures 6E1–E3). Those axons are not labeled with anti-GFP, but they co-label with anti-GABA (Figures 6E1 inserts). Some GFP stained mPNs do not show labeling with AmOA1 (Figures 6E2,E3), but they nevertheless co-stained with anti-GABA (Figure 6E2 insert).

#### A subset of local GABAergic interneurons in the drosophila antennal lobes label with anti-OA1 in the cell bodies, axons, and endings in the glomerulus

Anti-OA1 staining indicated cell bodies of neurons located laterally (Figures 6A,B,D). Scattered, stained processes are exhibited in all glomeruli and coarse areas of the antennal lobe. In the antennal lobe, GABA-like immunoreactivity is found in the LNs with cell bodies in lateral and dorso-lateral clusters. Furthermore, in addition to the two classes of GABAergic LNs, LN1, and LN2, there are GABAergic mPNs in the ml-APT tract. The LNs supply GABAergic processes in glomeruli (Figure 6F). Comparisons of anti-OA1 staining (magenta) with anti-GABA staining (green) in the antennal lobe suggest that OA1 is expressed in the subpopulation of local GABAergic interneurons (white) and in the GABAergic ml-APT multiglomerular PNs. The glomeruli are homogeneously stained with the anti-OA1 suggesting that all three types of GABAergic neurons express OA1. In addition, in the lateral cluster there are neurons marked by anti-OA1 but not by anti-GABA. These neurons, which are not GABAergic, might be excitatory LNs or non-GABAergic ml-APT PNs that connect antennal lobe to the LH (Tanaka et al., 2012a,b).

## Discussion

Several studies have documented the role of OA in driving plasticity linked to associative conditioning in fruit flies (Schwaerzel et al., 2003; Kim et al., 2013) and honey bees (Hammer, 1993; Farooqui et al., 2003). Our current study was motivated by an effort to understand the downstream components of OA signaling. That is, how OA induces neural plasticity by acting via different types of receptors. We identified neurons that express the AmOA1 receptor in neural networks of the antennal lobe (AL) of the honey bee and fruit fly. We also focused on the AmOA1 receptor because of its established role in behavioral plasticity in fruit flies (Kim et al., 2013) and because of the availability of a characterized antibody against this receptor (Sinakevitch et al., 2011). Our data show similar expression patterns in both species. Anti-AmOA1 receptor antibodies label subsets of GABAergic local neurons and mPNs. In addition, we show that AmOA1 receptors are expressed on non-GABAergic neurons in the antennal lobe. In contrast, we could not find such clear evidence that AmOA1 is expressed in ORN receptor terminals or uPNs.

How could this expression pattern account for changes in calcium responses of uPNs after associative conditioning (Fernandez et al., 2009)? Changes in calcium dynamics in uPNs are subtle and consist of many different patterns of shifts from excitation to inhibition and vice versa. OA binding to AmOA1/DmOA1 results in the release of calcium from cytosolic stores (Han et al., 1998; Grohmann et al., 2003; Beggs et al., 2011; Hoff et al., 2011), which would likely make GABAergic LNs and mPNs more excitable. Therefore, the plasticity reported in honey bee uPNs (Fernandez et al., 2009; Locatelli et al., 2013), in so far as it is induced by AmOA1, must have arisen indirectly by changes in the action of inhibition in the antennal lobe network. This conclusion is consistent with a recent study employing OA application to the honey bee antennal lobe (Rein et al., 2013) which produced both excitatory and inhibitory shifts in calcium dynamics of uPNs. This study also concluded that OA likely targets inhibition in the antennal lobe.

Clearly OA has multiple targets via AmOA1 in the antennal lobe. However, AmOA1 might not be the only modulatory pathway for OA. A second type of OA receptor (OA2) has been identified in fruit flies and honey bees (Evans and Robb, 1993; Roeder, 1999; Hauser et al., 2006). OA2 stimulates adenylate cyclase and thereby increases the concentration of adenosine 3′,5′-cyclic monophosphate (cAMP). Very little is known about the distribution of OA2 in the nervous system of either species.

**Figure 7:**
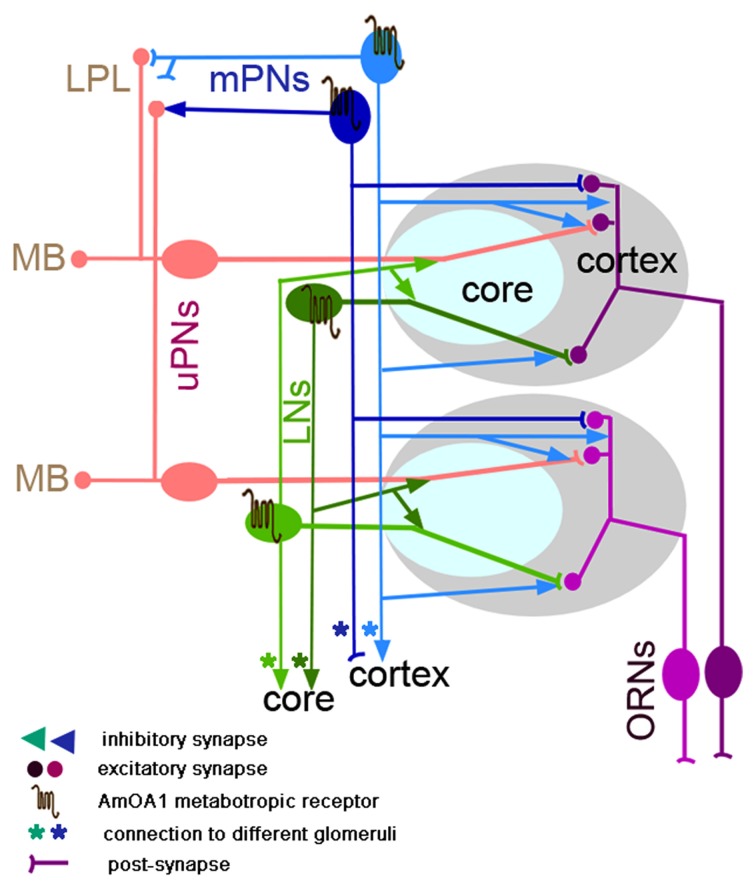
**Schematic representation of the neural network proposed for a glomerulus in the honey bee (model architecture based on Fonta et al., 1993; Abel et al., 2001; Nishino et al., 2009; Meyer and Galizia, 2012; Girardin et al., 2013).** Octopamine is released into the whole glomerulus in both core and cortex and acts on the GABArgic local interneurons (LNs) and on GABAergic multiglomerular projection neurons (mPNs). Each glomerulus has a uniglomerular PN (uPNs) that branches into both core and cortex areas of the glomerulus. uPNs receive excitatory synapses from ORNs in the cortex area and inhibitory synapses from hetero-multiglomerular local neurons from neighboring glomeruli in the core area. The uPNs also receive inhibitory synapses in the cortex that come from the mPNs. In the glomerulus there are GABAergic and non-GABAergic multiglomerular LNs. For simplicity, only one type of the GABAergic LNs is shown in the glomeruli; LNg (LN1 or heteroLN in Figure 1) that branches in all areas of the glomerulus (cortex and core) where it receives excitatory synapses from ORN in the cortex and inhibitory synapses in the core and sends the inhibitory output into the neighboring glomeruli. Both types of GABAergic neurons (LNs and mPNs) express AmOA1 receptors in the glomerulus. Based on our indirect evidence of AmOA1 and GABA stainings in glomeruli, we hypothesize two possible mPNs connections, one that receives excitatory synapses from ORNs in the glomerulus cortex and makes synapses in the LH as well as another that receives synaptic input from LPL and LH and makes synapses onto the processes in the cortex. Future physiological and anatomical studies will clarify the branching patterns that we proposed for mPNs.

Figure 7 summarizes our findings and presents a model incorporating our information about AmOA1 in antennal lobe glomeruli exclusively for the honey bee. In order to construct the model, we present the distribution of AmOA1 receptor in a more detailed discussion of important neuroanatomical features revealed in our current study.

### Olfactory receptor neurons (ORNs)

In the fruit fly, ORNs that express the same receptors project to the same glomerulus (Laissue and Vosshall, 2008; Tanaka et al., 2012a). Although it has not been directly shown, we assume that this is also true for the honey bee. However, there are some differences in the structure of the glomeruli between fruit flies and honey bees. In the honey bee the endings of ORNs are clearly functionally and topologically organized in the outer layer (cortex) of the glomerulus (Nishino et al., 2009). This was not the case in fruit flies in which the distribution of ORNs has no precise topological organization within the cortex. This difference might be due to a somewhat different organization of the antenna combined with inputs from the MP. The core area of glomerulus is free from ORNs in both insects (Hummel and Zipursky, 2004). We found no indication of AmOA1 expression in ORN terminals.

### Uniglomerular projection neurons (uPNs)

uPNs reveal dendritic branching limited to a single glomerulus, and they project axons to higher order neuropils (MB and LPL) (Abel et al., 2001; Kirschner et al., 2006; Galizia and Sachse, 2010). In both insects, the dendrites of uPNs branch throughout the entire glomerulus (both cortex and core). In fruit flies, the ORNs synapse on uPNs in each glomerulus (Wilson, 2011). In the honey bee, there has to date been no conclusive study to show that ORNs synapse onto uPNs. Nevertheless, for our model in Figure 7 we assume that uPNs are postsynaptic to ORNs in the cortex area of the glomeruli, and they can be inhibited by LNs in the core and send information to the MB and LPL.

In the honey bee, approximately five uPNs branch in each glomerulus (Rybak, 2012). Thick fibers are located at the entrance of the core area and fine fibers in the cortex, which could be related to the possibility that uPNs receive different types of inputs to these two areas. Consistent with other reports, we also observed one fine branch that extends from the uPN to the neighboring glomerulus cortex (not shown in Figure 7).

In both insects, the uPNs form two pathways that connect the antennal lobe with the calyx and LH via the m-APT and l-APT tracts (Okada et al., 2009; Galizia and Rössler, 2010; Tanaka et al., 2012a), respectively. Furthermore, the axon terminals from each pathway are topographically organized in the calyx and LPL for both insects. uPNs do not express anti-AmOA1 receptors in their branches within glomeruli or in their axons in the calyx, LPL, and LH.

### Multiglomerular PNs

Both fruit flies and honey bees have multiglomerular PNs. mPNs are GABAergic in fruit flies and have postsynaptic sites in the glomerulus with presynaptic sites in the LH (Okada et al., 2009). In the honey bee, there are at least 17 GABAergic ml-ACT mPNs that branch in different areas of the LH, LPL, and LH. We cannot, at this point rule, out the possibility that there could be two kinds of mPNs in honey bees, which we represent in the schematic in Figure 7. Some mPNs (dark blue) might receive input from ORNs or one or more types of antennal lobe LNs in the cortex of multiple glomeruli and synapse in the LPL and LH, as in fruit flies (Okada et al., 2009). Other mPNs (light blue) might receive input in the LPL and LH and synapse in the glomerular cortex. The latter subtype of mPN, if they exist, would constitute a feedback pathway to the antennal lobe. A more detailed analysis of the GABAergic mPNs will be needed to test this hypothesis. We can, however, conclude that subsets of GABAergic mPNs express AmOA1 in processes in the LPL and LH.

### Local interneurons (LNs)

Large numbers of LNs have been reported in the honey bee (Galizia and Sachse, 2010). Similarly, a high diversity of LNs has been reported in the antennal lobe of fruit flies (Chou et al., 2010): ~100 ipsilaterally projecting and ~100 billaterally projecting LNs. Most of the LNs are GABAergic (Ng et al., 2002; Wilson and Laurent, 2005; Okada et al., 2009; Seki et al., 2010), but some LNs are excitatory (Olsen et al., 2007b; Shang et al., 2007; Seki et al., 2010) and cholinergic (Shang et al., 2007; Das et al., 2008). The most ventral bilaterally projecting LNs are glutamatergic LNs (Chou et al., 2010). These LNs are also diverse in their glomerular innervation pattern, fine dendritic structures, densities and distribution of presynaptic terminals, and odor response properties (Okada et al., 2009; Chou et al., 2010; Seki et al., 2010; Tanaka et al., 2012a). In the honey bee, the LNs are also reported to have a high diversity in their dendritic distribution and response properties (Schafer and Bicker, 1986; Fonta et al., 1993; Meyer and Galizia, 2012; Girardin et al., 2013). In addition to GABAergic LNs, the honey bee has a large number of histaminergic LNs (Dacks et al., 2010), which have not been reported in fruit flies.

In the honey bee antennal lobe, we found approximately 375 GABAergic LNs. A previous study of GABA in the honey bee antennal lobe reported 750 GABAergic neurons (Schafer and Bicker, 1986). The difference between our study and that one might be due to counting GABAergic neurons in clusters that are dorsal to the antennal lobe. In our study, injection of dye into the antennal lobe combined with subsequent anti-GABA staining did not co-label the clusters of cells that are located rostrally.

Our results show that in both insects GABAergic and non-GABAergic neurons express AmOA1. The majority of the GABAergic LNs express the receptor in their branches throughout the glomeruli. In the model (Figure 7) we only consider heteroLNs, which are a subpopulation of LNs that have extensive arborizations in the core and cortex of one glomerulus and then less dense arborizations in the core of many glomeruli (Fonta et al., 1993). Our hypothesis is that heteroLNs receive excitatory input from ORNs in the cortex and inhibit the neural circuitry located in the core of neighboring glomeruli (Figure 7). We consider another subpopulation of LNs, the homoLNs (GABA and non-GABAergic), which make intraglomerular connections. These LNs receive inhibitory input from heteroLNs in the core and relieve the inhibition in other glomeruli. Among the homoLNs in the honey bee, there are 20 alatostatin GABAergic neurons that branch in the core and inner area of the cortex of the glomerulus (Kreissl et al., 2010). HeteroLNs might inhibit homoLNs in the core of neighboring glomeruli and thus provide additional excitation for uPNs in the given glomerulus.

### The glomerulus as a functional unit of coding and plasticity

We found that glomeruli in the antennal lobe of the honey bee, independent of their size, all have a similar ratio of cortex to core. The neurons that are exclusive to each glomerulus are uPNs and ORNs. Both of these types of neurons are excitatory and their properties are not directly modified by OA via AmOA1. On the other hand, GABAergic LNs interconnect glomeruli and thus shape signals from uPNs and possibly ORNs. Each of the mPNs provides feed-forward (to the LPL) or possibly feedback (from the LPL) inhibition involving a large number of glomeruli. According to our model (Figure 7), the core of the glomerulus might be a computational unit that processes information from the ORNs and from other glomeruli while influencing the response profile of uPNs. This affects the information flow to the calyx of the MB and to the LPL and LH. Reinforcement learning through activation of AmOA1 receptors would shape this processing by targeting GABAergic processing.

### Hypothesis for reinforcement-based plasticity via AmoA1

We propose a model for antennal lobe plasticity based on OA release onto AmOA1 receptors. In the antennal lobe, the fibers of the VUMs branch mostly in the cortex of the glomerulus with varicosity-like distributions (personal observation; Sinakevitch et al., 2005, 2011; Sinakevitch and Strausfeld, 2006). These wide branching fibers in the cortex could modulate responses of the ORNs, uPNs, and LNs during olfactory conditioning. We hypothesize that OA release from VUM is contingent upon gustatory stimulation of sucrose receptors on the mouthparts coincident with cholinergic input from ORNs to prime OA release. This hypothesis is based on pharmacological evidence of different types of acetylcholine receptors expressed in DUM neurons (Lapied et al., 1990; Grolleau et al., 1996; Sinakevitch et al., 1996; Courjaret and Lapied, 2001), which are homologs of VUM neurons (Sinakevitch et al., 2005; Sinakevitch and Strausfeld, 2006). Second, OA release from VUM acts on AmOA1 receptors expressed in GABAergic and non-GABAergic LNs, which leads to the prediction that OA will increase the excitability (Riffell et al., 2013), leading to increased inhibition in the cortex of the glomerulus. OA will not have that effect on other neurons, for example PNs, which may or may not express another type of OA receptor AmOA2 (Hauser et al., 2006) that does not induce immediate excitability. Future empirical and modeling studies need to test this hypothesis.

### Conflict of interest statement

The authors declare that the research was conducted in the absence of any commercial or financial relationships that could be construed as a potential conflict of interest.
